# Review of approaches to the use of unmanned aerial vehicles, remote sensing and geographic information systems in humanitarian demining: Ukrainian case

**DOI:** 10.1016/j.heliyon.2024.e29142

**Published:** 2024-04-04

**Authors:** T. Hutsul, M. Khobzei, V. Tkach, O. Krulikovskyi, O. Moisiuk, V. Ivashko, A. Samila

**Affiliations:** a Department of Geomatics, Land and Agromanagement, Yuriy Fedkovych Chernivtsi National University, Kotsyubynsky 2, 58002, Chernivtsi, Ukraine; b Department of Radio Engineering and Information Security, Yuriy Fedkovych Chernivtsi National University, Kotsyubynsky 2, 58002, Chernivtsi, Ukraine; c Department of Computer Sciences, Yuriy Fedkovych Chernivtsi National University, Kotsyubynsky 2, 58002, Chernivtsi, Ukraine; d Integrated Center for Research, Development and Innovation in Advanced Materials, Nanotechnologies and Distributed Systems for Fabrication and Control, Stefan cel Mare University of Suceava, Street University 13, 720229, Suceava, Romania; e Faculty of Electrical Engineering and Computer Science, Stefan cel Mare University of Suceava, Street University 13, 720229, Suceava, Romania

**Keywords:** UAV, GPR, GIS, Remote sensing, Artificial intelligence, Demining

## Abstract

The history of the use of mines dates back almost two centuries. The geography of their use and the associated social harm have made them, without exaggeration, a global problem. At the same time, searches were underway for safe methods of their neutralization using various technical means. In so doing, until now, none of the existing methods provides a 100% guarantee of cleaning the territory, which determines the purpose of finding innovative methods and the possibility of combining them with existing ones. Unmanned aerial vehicles (UAVs) are becoming a real modern breakthrough in the field of intellectual achievements. Obtaining optimal results when solving a wide range of different problems, together with the development of composite materials, software, and the latest navigation equipment, make the tasks assigned to them and the expected results more and more difficult. UAVs allow people not to be in life-threatening conditions, to conduct activities beyond their physiological and psychophysiological abilities. The combination of the ability to collect spatial data during flight in various ranges of remote sensing with the possibility of carrying variants of useful equipment opens up prospects for their use in the field of demining territories. Supplementing UAV technologies with modern information systems for processing and analysis of information (expert systems, machine learning, computational intelligence, distributed artificial intelligence, neural networks, etc.), including spatial geographic information systems (GIS), opens up great prospects in the field of humanitarian demining of territories.

## 1 Introduction

According to the press service of the State Emergency Service of Ukraine (SESU), as a result of the Russian military invasion, as of mid-November, about 30% of the territory of Ukraine was mined [1], and by the beginning of 2023, more than 40% [2]. The expected duration of complete humanitarian demining of the territory is from 5 to 10 years. During this time, mined territories will significantly limit the possibilities of safe movement in them, active use in economic processing, or as a spatial basis, will slow down significantly the pace of economic development and restoration of Ukraine.

**Fig. 1:**
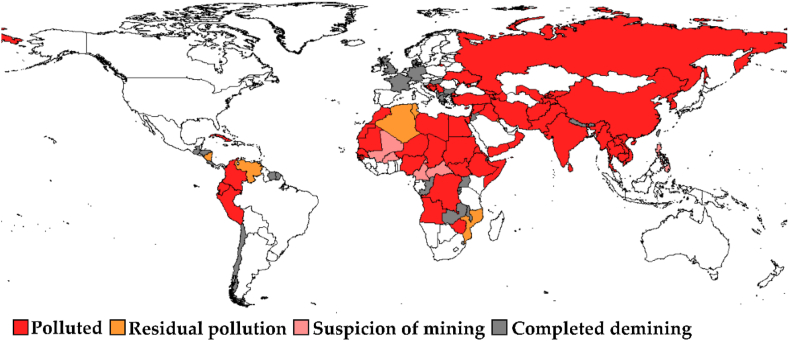
Pollution with anti-personnel mines (as of 2022).

The problem of demining territories affected by the consequences of hostilities is a world-level problem (Fig. 1), which requires urgent innovative approaches to its solution. Approximately 110 million mines remain in about 60 countries of the world, including Ukraine, even decades after the end of the wars. 1.9 million new mines are installed annually, and only about 100 thousand are removed [3].

Fear of landmines « … contributes to the refusal of refugees to return to their homes, reduces access to fertile agricultural regions, delays the restoration of infrastructure, delays the provision of public services and interrupts activities that contribute to the inflow of foreign currency into the budget, such as tourism » [4].

**Fig. 2:**
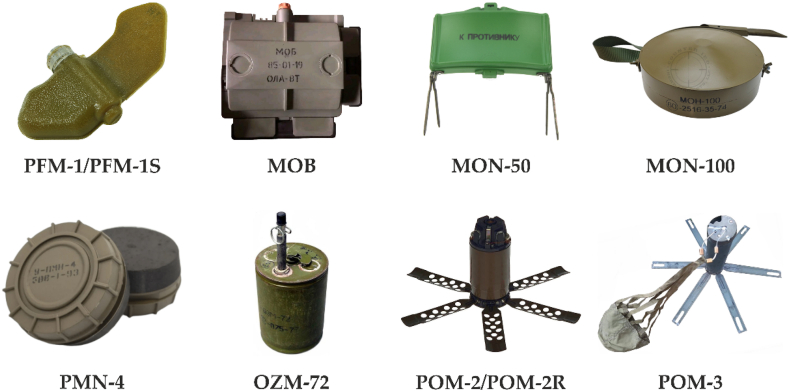
Common types of anti-personnel mines on the territory of Ukraine.

Since February 2022, Russian troops in Ukraine have used [5] at least 8 types (Fig. 2) of anti-personnel mines – PFM-1 PFM-1S; MOB; MON-50; MON-100; OZM-72; PMN-4; POM-2/POM-2R; POM-3.

The cost and time required for demining is much greater than for the manufacture and installation of mines. This motivates both governments and the scientific community to find safe, fast and accurate demining solutions.

## 2 Materials and methods

There are many methods for detecting explosives and landmines (see Table 1). Most of them are limited in sensitivity and/or operational complexity due to terrain, climate and soil obstructions, such as splinters and stray metal fragments, which create numerous false alarms and slow detection rates to an unacceptable level. The classification of detection methods based on the physical principle of interaction with unmasking features and separately from the information and measurement tools and platforms on which these tools are located is proposed in Ref. [6], and includes: 1) mechanical; 2) electromagnetic; 3) chemical; 4) magnetic; 5) acoustic.

**Table 1:** Modern landmine detection methods [[7], [8], [9], [10], [11], [12]].

Landminedetection methods	Types of landminedetection methods	Earth remote sensing methods
Biological	animals: *dogs, pigs, rodents, etc.*	
	insects: *ants, bees, etc.*	
	plants: *sugar beet, lettuce, etc.*	
	bacteria	
Electromagnetic	metal detectors	
	ground penetrating radar (GPR)	☑
	microwave radiometry	☑
	radiometry of millimetre waves	☑
	electroimpedance tomography	☑
	infrared radiation (IR)	☑
Optical	visible radiation	☑
	lidar	☑
Chemical	mass-spectrometry	☑
	infrared reflection (IR) absorption spectroscopy	☑
	optoacoustic spectroscopy	☑
	combination scattering	
	іimmunochemical sensors	
	electronic noses	
Nuclear	nuclear quadruple resonance (NQR)	
	based on neutrons	
Acoustic	sound and ultrasound	
	transition of acoustic data to seismic data	
Mechanical	tool tunnelers	
	cleaning machines	
Artificial intelligence	machine learning	
	expert systems	
	computational intelligence	
	distributed artificial intelligence	
	smart interfaces	
Geoinformation methods	cartometric operations	
	selection operations	
	reclassification	
	cartographic algebra	
	statistical analysis	
	spatial analysis	
	overlay analysis	
	network analysis	
	geostatistical modeling	
	spatial interpolation	

None of the currently developed methods for detecting landmines and explosive devices using specialized technical means in terms of such important parameters as distance, sensitivity, selectivity and speed, satisfies either the requirements of the current UN standards on humanitarian demining, or the general need for global and operational demining planet [13].

Until now, the main devices for detecting explosive objects are still electromagnetic (EM) metal detectors, the first prototypes of which were used during the Second World War. Metal detectors of this type have a number of disadvantages. Due to the fact that the EM signal reflected from the anti-personnel mine is much weaker, the sapper must increase the sensitivity of the detector. At higher sensitivities, many more other metal objects, such as nails and hull debris, interfere with the detector. Despite the addition of electronic filters to reduce “ground noise”, there are still many problems with this technology. The sapper performs a very delicate extraction with a pointed stick to classify the source of the reflected signal: a landmine, tampering or just a rusty nail. In this sense, finding mines is easy enough, however, separating them from other objects is difficult and extremely dangerous. Another significant disadvantage of EM metal detector is the inability to detect explosive objects in non-metallic containers (mines, hand luggage, plastic containers, etc.).

The use of ground demining systems causes significant risks associated with damage to special equipment and, most importantly, increases threats to the lives of personnel. UAV technologies are recognized by the UN Mine Action Service as a real tool [14].

One of the latest approaches to the use of unmanned aircraft to solve demining tasks is based on the use of mine detectors installed on UAVs as a payload. Another approach consists in the use of multispectral equipment installed on a drone for conducting reconnaissance of the mine situation and detecting mines. Still another approach is to use infrared equipment mounted on the drone and its response to the temperature difference between the mine and the surface of the terrain.

The purpose of concepts [6] aimed at achieving high productivity and reliability of search, detection and recognition of mines for their further disposal is to use multi-functional fleets of UAVs equipped with various information and measurement tools, whereas the implementation of such a list of tasks for a single device seems almost unrealistic.

**Fig. 3:**
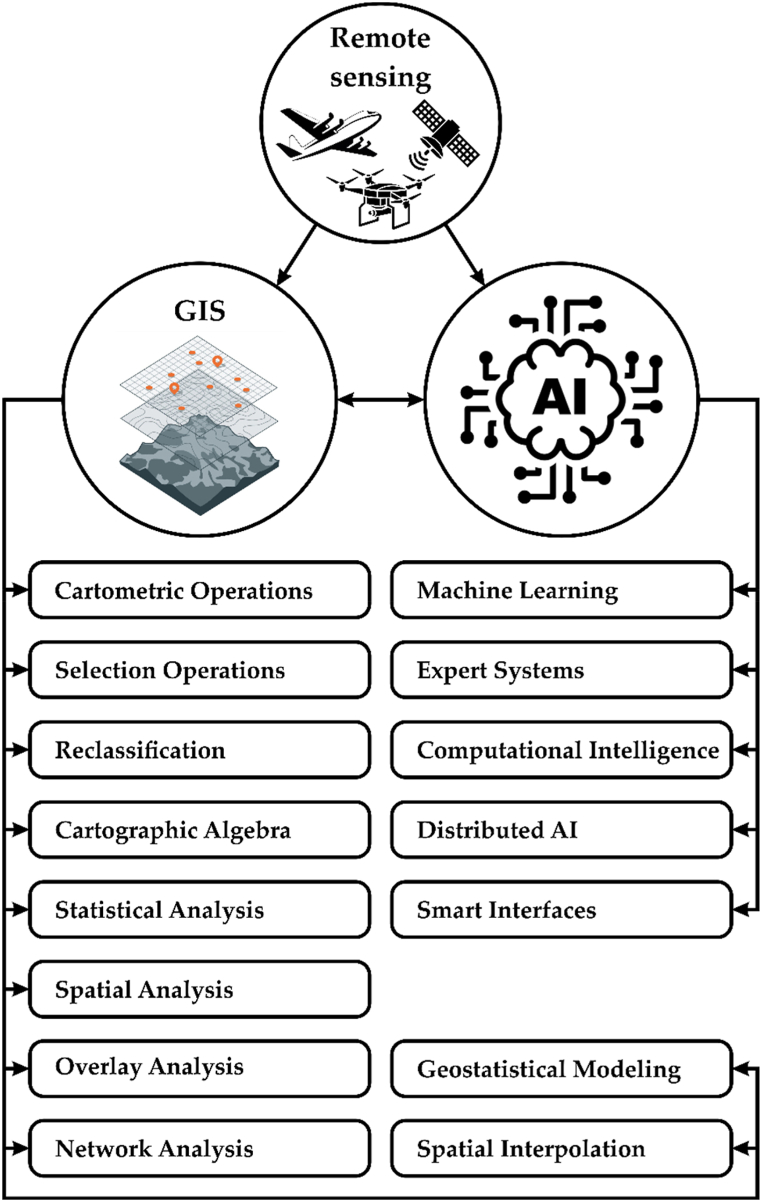
Possibilities of combining GIS methods and artificial intelligence technologies. AI – Artificial intelligence; GIS – geographic information systems.

The development of related information technologies in most cases, in particular, GIS and artificial intelligence technologies (Fig. 3), is practically completely aside, and is auxiliary, although definitely promising.

Most UAV technologies are implemented separately, but their combination and interaction will improve the demining process, moving from experimental prototype tests to specialized field tests.

## 3 Current methods, technologies and data sources for demining

An effective demining solution must take into consideration that explosive substances must be detected and neutralized before they explode. Most of the existing methods have significant drawbacks, such as cost, efficiency and accuracy, and establishing optimal trade-offs between them is still not possible. UAVs, GIS technologies, remote sensing and artificial intelligence are comprehensively capable of revolutionizing traditional approaches to the detection and disposal of landmines.

### 3.1 Unmanned aerial vehicles and remote sensing

**Fig. 4:**
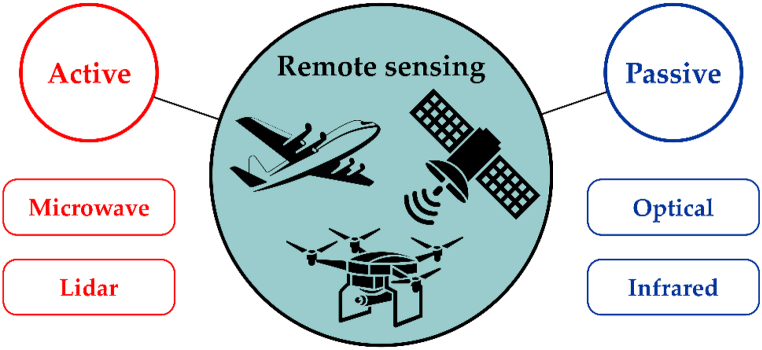
Classifications of modern remote sensing methods for collecting geospatial data (according to Ref. [16]).

Traditionally, methods of remote sensing include methods that allow obtaining information about objects on the Earth's surface, phenomena and processes from space or air and are based on non-terrestrial registration of electromagnetic radiation of the Earth's surface in various spectrum ranges (Fig. 4) [15].

Depending on the height of shooting of the territory, the following are distinguished: space, aerial shooting and shooting by unmanned aerial vehicles (UAVs). The use of UAVs, as opposed to land-based drones, provides greater space navigation capabilities combined with mine detection safety. UAVs can work autonomously, more efficiently and with a lower percentage of errors. A UAV can be used as a platform for collecting remote sensing data about the areas it accompanies. Thus, it collects information related to an event or object on the Earth's surface without physical contact.

The UAV/Satellite synergy (Fig. 4) is important for understanding the dynamics of changes on the Earth's surface. On the one hand, these two data sources create significant amounts of data contributing to the Big Earth Observation. On the other hand, each system has specific shooting features [17], which are the result of a trade-off between resolution (spatial, spectral and temporal), line of sight and signal-to-noise ratio.

The creation and use of UAVs became a major breakthrough in the field of intellectual achievements [18]. Innovation is evident in all elements: from modern advances in avionics and propulsion systems, the growth of battery capacity, the introduction of composite materials, the latest navigation equipment and software [19].

Recent advances in telecommunications technologies, such as 5G and the Internet of Things (IoT) play a critical role in aerial communications using UAVs. Depending on the region of operation, UAVs can be used to expand the network coverage and bandwidth of wireless networks beyond 5G. In such cases, UAVs also play the role of intermediate nodes or flying base stations. This helps to conduct operations in hard-to-reach places, and the integration of cloud computing with UAVs increases their role by providing additional computing power [20]. We are entering the era of «big data » accelerated by UAVs that transmit large data packages from an on-board camera to the network [21].

They can be equipped with multiple sensors, including cameras, inertial measurement units (IMU), LiDAR and GPS [22,23], for real-time acquisition and transmission, and carry payloads/other equipment on board. Multispectral, hyperspectral, thermal infrared cameras, radar systems, etc. are used with UAVs to obtain images of the surveyed territory [[24], [25], [26]].

The main device with which it is possible to determine the location of mines on the Earth's surface from a height using direct de-encryption features is a high-resolution camera. Partially buried mines can be observed if part of the mine is visible from the air. Detection of mines is based on atypical elements in the fields, namely tire tracks, dry grass on a green surface, unusual traces of movement on the grass, sudden breaks in colour, texture, content, shape and size. Such cases, with a high probability, testify to the carrying out of works on mining the territory.

The use of stereoscopic variants of photo images provides additional information due to the possibilities of visualizing the terrain in three-dimensional space [27]. The interpretation of the received images can be done by humans or with the help of appropriate algorithms.

The role of unmanned aerial vehicle technologies has expanded from the usual technical survey of the terrain to surveying the fields with a variety of sensors before demining [28].

The first UAV for humanitarian demining came from the EU ARC project [29]. Over the past 5–10 years, the use of UAVs with visible colour sensors for humanitarian demining has increased.

UAVs can collaborate with other robotics [30] creating a 3-D terrain map from photographs in a shared point cloud [31]. Such cooperation is especially useful in a cluttered environment. Testing of this method was carried out on a real construction site and allowed to obtain promising results.

Infrared (IR) landmine detection technology uses the difference in IR radiation caused by mines laid just below the surface to detect them. A mine always has a different thermal mass than the surrounding ground. This is especially noticeable in places of damaged and undisturbed soil (recently buried mines). This makes such radiation pronounced at peak times of the day, when the temperature difference is most noticeable. This technology is more dependent on soil cover than other methods. With additional ground cover information, IR systems can even distinguish between different types of landmines. Infrared radiation consists of a wavelength from 0.7 μm to 1.0 mm in the optical range.

One of the first studies [32] on the detection of landmines using IR waves was carried out by the Defence Research and Development Canada (DRDC) and Itres Research in 1978–1989. The project was based on a hierarchical image processing algorithm to detect sparsely distributed bright area with a width of several pixels in a monochromatic image.

In 2015, TELOPS, a Canadian research company specializing in infrared and hyperspectral imaging, demonstrated the possibility of detecting buried objects using an airborne LWIR hyperspectral scanner [33]. Thermal hyperspectral images of areas containing previously buried man-made objects were obtained from the aircraft platform. They found that the disturbed soil directly above the buried mine was warmer than the undisturbed area next to it.

The method of mapping minefields using centimetre resolution images from a helicopter-type UAV equipped with a multispectral camera and a thermal infrared camera is given in Ref. [34]. This method was tested in areas with actually buried landmines.

In an effort to address the problem of PFM-1 mines, researchers at Binghamton University [35] developed a protocol based on remote sensing of the unique heat signatures associated with the PFM-1 and the aluminium cartridge case. The body and wing of the mine heat up faster during early heating under direct sunlight. Individual PFM-1 anti-personnel mines, which are invisible to direct sunlight, heat up quickly and can be detected on both visible and thermal images by their distinct shape and thermal properties. During field tests, researchers were able to successfully identify all elements of a randomized minefield covered with PFM-1 mines from a height of 10 m with a thermal camera mounted on an inexpensive commercial UAV.

Although this methodology cannot completely replace traditional manual demining to unequivocally declare an area mine-free, the PFM-1 remote thermal field detection allows accurate assessment of minefield presence, orientation, and any overlap between two or more minefields. Available inexpensive commercial UAV platforms equipped with thermal imaging cameras allow accurate assessment of minefield presence, orientation and potential minefield overlap.

To date, there are no automated solutions for the detection of surface unexploded ordnance using thermal imaging. During a project in Bosnia and Herzegovina in 2019, Norwegian Public Aid collected data on unexploded ordnance and made it available for research [36]. Thermal images with a size of 720 × 480 pixels were collected using a UAV at a height of 3 m. The involvement of artificial neural networks has proven its effectiveness [37], and during image processing, it has been possible to achieve a probability of identification at the level of more than 90%.

Hyperspectral sensors with the ability to register an electromagnetic field in a wide range of electromagnetic spectra expand the idea of visual observation and provide an order of magnitude more accurate information. This results in a reflection spectrum value for each pixel in the image, which helps to identify the components in the image. The first cameras were broadband, and recorded the intensity of light in a wide range of the spectrum. However, the development of sensitive photodetectors made it possible to obtain slices of images in narrower bands. This increased the spectral capability of cameras and increased the number of materials that could be distinguished.

The availability of hyperspectral sensors suitable for use with multi-engine UAVs has enabled the development of system for humanitarian demining. Though large and expensive airborne hyperspectral systems have been available for over two decades, they have had little impact on humanitarian demining [38].

The US Army launched the Hyperspectral Mine Detection Phenomenology Program (HMDP). The main objective was to determine the existence of spectral characteristics useful for landmine detection [39].

It was possible to collect high-quality hyperspectral signatures of background materials and mines, measure temporal effects on buried landmines and measure a statistically significant set of hyperspectral signatures of surface and buried mines in natural soils under controlled variable variations. The results of the spectral analysis obtained during the recordings of the HMDP project are presented in Ref. [40].

The work [41] describes the first experiment aimed at detecting landmines using an airborne hyperspectral imaging system in real time. First, radiometric data correction is applied to the raw data. Subsequently, spectral classification algorithms are applied to the corrected data. The spectral signature library provides reference spectral vectors. Classification results are stored and displayed in real time.

Geo-penetrating radar systems (GPR) is a geophysical method that is based on the principles of reflectometry and allows the detection of buried objects and objects in various environments, including soil, rocks, ice, fresh water, pavements and various structures. Difficulties in detecting small amounts of metal in plastic mines contributed to the emergence of this technology. Compared to metal detectors, the detection depth range is significantly expanded and risks are minimized. The method uses electromagnetic waves of the microwave range (UHF/VHF frequencies) of the radio spectrum and detects signals reflected from the Earth's surface with the help of a receiving antenna. Soil moisture significantly affects the possibility of detecting mines, therefore it is recommended to carry out scanning work when its amount is minimal. GPRs are calibrated for use on different types of soil. The most common systems using GPR are portable means, ground vehicles, and more recently UAVs [42] or various combinations. In Ref. [43] it is proposed to make small GPRs weighing up to 1.1 kg for installation on UAVs.

Studies [[44], [45], [46]] are based on the integration of a GPR radar system with an SDR radio communication system of an unmanned aircraft as a dynamic object. However, to date, an acceptable GPR efficiency is possible only if the UAV flies at a minimum height above the Earth's surface, with regard to wind disturbances. An example of integration of a pulse radar system with a UAV and simultaneous generation of a spectral map in real time is considered in the study [47].

A lightweight GPR system with low power consumption, operating on the principle of continuous step frequency radar, has been developed [48]. The results of the experiments showed that the system is capable of detecting both metal and plastic buried targets. However, this is achieved only under the condition of a low flight height of the UAV over the mined area up to 0.5 m.

In the study [49], it is noted that GPR accuracy exceeds 80% in tested scenarios. However, a number of conditions must be met, namely the artefact must occupy an area >60 cm^2^, and at least 30% of its material consists of metal.

To increase the probability of detecting mines, it is recommended to install a magnetometer in a pendulum manner under a UAV [50] to minimize the distance between the Earth's surface and the magnetometer. The laser altimeter is optimized to ensure stable drone flight at low altitudes by accurately calculating altitude even in dusty and bushy environments.

### 3.2 Geoinformation systems and technologies for demining territories

Most mine action programs have a national authority and a mine action centre that uses advanced GIS-based information management systems. One technology that is not always associated with the complex process of detecting buried landmines, but which nevertheless gains prominence in demining circles, is GIS.

Interest in GIS lies in the use of this technology as a spatial database for mine-affected area surveys, and in the potential of the technology to coordinate demining efforts at the national and international levels. Mine action database and geographic information system efforts are being shared with other information collection sectors, greatly expanding the potential for further data analysis. In most cases, the potential application of geomatics technology has been reduced to its use in multi-sensor detection systems. The growing interest in GIS is associated with their rapid development, as well as the creation of separate sessions at demining conferences devoted to GIS technologies.

The diversity of anti-personnel mines and minefields, as well as the complex way in which they are distributed in individual countries and regions, has led to calls for systems that can collect, display and process spatial mine data.

Despite advances in surveying and mapping technology in recent years, basic mapping is inadequate or non-existent in some parts of the world. This is especially true in war-torn countries [51].

The initiation of demining activities includes a socio-economic impact study to identify areas likely to be affected by mines, and where further demining would have the greatest impact on the local population. This is mainly due to the factor of population density and/or the agricultural potential of the territory. The pre-demining activity, which consists in collecting data and information about the approximate location of minefields, accident sites, is known as « non-technical survey». This demining activity has been greatly enhanced by satellite and aerial surveillance technologies, modern GIS, such as ArcGIS, and Google Earth services [52].

One of the key issues of humanitarian demining is the selection of areas for clearance. Using GIS, aerial photography and satellite data in combination with GNSS during a non-technical survey, all available and additionally collected data can be georeferenced into an information system [53]. GIS methods can be used to integrate data from the history of hostilities (chronology of changes in the defensive lines of the battlefield during the years of the conflict) for the purpose of their synthesis and interpretation [54]. Remnants of war identified by remote sensing data, called « visual cues», may indicate the presence of mines or explosive devices in the immediate vicinity.

There is an increasing number of tests of GIS methods in combination with geostatistical methods for modeling mine risks in order to supplement data on demining activities [55]. GIS are integrated into humanitarian demining with different goals.

One of them is modeling the ability of a mine-affected community to adapt to landmine pollution [56], creating risk maps that outline high-risk areas that require priority demining [57].

All activities and data collected during demining are stored in the GIS and used to support expert decision making in mine action centres. A decision support system (DSS) is based on a combination of GIS analysis and a multi-criteria method to ensure effective mine action management. GIS is a powerful tool for generating aggregated information used in multi-criteria analysis, as a well as a connecting link between hierarchical levels of decision-making in DSS.

Any development of a support and decision-making system will in one way or another face a set of criteria [58], and will deal with geodata in the form of satellite images or specific coordinates of objects determined by GNSS. The absence of a spatial analysis component requires an unambiguous transition to GIS systems.

The development of a decision support tool based on a geographic information system is illustrated by references to case studies in Afghanistan and Western Sahara. The successful use of a GIS-based decision support system has led to the development of a spatial multi-criteria analysis tool [52].

The results of the DSS pay the way for the creation of a series of thematic mine danger maps. Mine danger maps are synthetic thematic works resulting from the synthesis of available data and expert knowledge. Their formation is intended to help end users identify priority scenarios for the reduction of mined areas and prioritize their clearance.

### 3.3 Possibilities for combining technologies for demining territories

In Belgium, a research project [59] focused on the use of data merging from multiple sensors (radar, metal detector and infrared sensor). The authors relied on the potential of multi-sensor data merging to optimize the detection of landmines at close range and reduce the area of mined territory. Simulation and merging of selected features are based on the theory of the likelihood function and the theory of probability. After simulation, data merging was performed in two stages. At the first stage, all data measured by one sensor was analysed. At the second, the results of three sensors were combined. The data synthesis ended with an identity decision, which the authors of the project left to a human observer with work experience.

FOI, the Swedish Defence Research Agency, was working on a project for the Swedish Armed Forces called the Multi-Optical Mine Detection System (MOMS). The goal of the project was to detect landmines using a combination of optical sensors. According to the authors, hyperspectral imaging is a promising option for automatic detection and recognition of open and semi-hidden mines, when a priori knowledge of the target's spectral signature is available. However, detection performance is limited when targets are camouflaged by natural vegetation or hidden by other objects. The authors state that no single configuration can provide the performance required under all operating conditions, and the choice of specific sensors and algorithms will depend on the environment and operating conditions [60].

Whereas expert systems try to feed human experience into a computer system, neural networks try to create meaningful models from small amounts of data. Neural networks recognize patterns that are not very clear to humans and adapt them to receive new information.

Neural networks, which are currently the standard for detecting and classifying objects in the field of remote sensing, began to appear as a supplement to remote sensing in 2009.

A key characteristic of neural networks is that they learn. A neural network program is initially given a data set consisting of many variables associated with a large number of cases or outcomes in which the latter are known. The program analyzes the data and processes all correlations, then again selects a set of variables that are strongly correlated with the known results as an initial model. This initial model is applied to try to predict the outcomes of different cases, and the predicted outcomes are compared to known outcomes. Based on this comparison, the program replaces the model by adjusting the parameters of the variables or even changing them. The neural network program repeats this process many times, trying to improve the predictive ability while improving the model. When the possibility of further improvement is exhausted in this iterative approach, the program is ready to predict the outcome of future cases.

As soon as a new large number of cases becomes available, this data is also fed into the neural network and the model is adjusted once more. A neural network is trained mainly on causal models from additional data, and its predictive ability is improved.

Of all the interesting properties of artificial neural networks, none captures the imagination as much as their ability to learn [61]. The training capabilities of artificial neural networks are limited, and there are many difficult questions to be determined. However, convincing achievements are already being made.

Since 2012, neural networks have outperformed other machine learning methods and have been successfully used in thousands of applications for classifying and detecting remote sensing objects [62].

Geospatial data provider PSMA Australia announced the release of Geoscape program in 2017. Its purpose is to obtain accurate spatial data automatically. So far, the first comprehensive dataset has been produced for Adelaide and rural South Australia, Canberra and the Australian Capital Territory. Geoscape data is generated using a new scalable approach for analyzing high-resolution DigitalGlobe satellite imagery using machine learning. These algorithms are self-learning, and already allow to detect building materials and their characteristics, the height of vegetation cover and its change over time, etc. The datasets resulting from the analysis will be continuously updated as new high-resolution WorldView satellite images become available.

Advanced classification and image recognition using machine learning/artificial intelligence has been used to identify mine probable areas [63].

Machine and deep learning algorithms have recently gained a high level of support in various UAV-related applications, such as resource allocation, obstacle avoidance, tracking, path planning, and battery life. Moreover, the availability of accurate data will facilitate the UAV's precision control, trajectory planning and survey tasks [64].

In [65], an effective combination of a UAV design with a magnetometer installed on it is considered. The complexity of use is related to the minimization of energy consumption. Involving deep machine learning with various modifications achieves 21–37% better performance than Synthesized Array Radar (SAR), neural network (CNN) and Double Deep Recurrent Q-network (DDRQN).

Several applications of deep learning in UAVs are considered in Ref. [66]. By adding different cameras to a UAV, different types of images can be obtained for further processing.

An example of a successful combination of UAV technologies and machine learning is given in Ref. [67]. The involvement of the R–CNN algorithm to automate the process of detection and classification made it possible to reach a level of 71.5%. Subsequently, the set of raw data was expanded, and the accuracy of detection of PFM-1 anti-personnel mines by UAV-based visual images (RGB) increased to 91.8%.

The performance of neural networks is also impressive. In the study [68] it took 1.87 s to detect scattered PFM-1 mines on a 10 × 20 m minefield. Accordingly, it would take 2 h 36 min to survey 1 km^2^ of territory. At the same time, the initial accuracy of identification of landmines was 71.5%. However, in order to increase the effectiveness to the level of 99.3%, the researchers identify several possible options. The amount of data for training and testing will be increased and diversified in terms of environmental conditions, orientation of mines in three-dimensional space and the addition of obstacles. The datasets covered by a UAV will also be automatically augmented by sharpening, rotating, cropping and scaling using various software.

In recent years, computational intelligence methods have been used to solve optimization problems. An innovative direction in the development of artificial intelligence methods is multi-agent methods of intellectual optimization that simulate the collective behaviour of animals [69]. These methods allow groups of individuals to solve various difficult practical problems in nature, which indicates the effectiveness of their behaviour, and hence the effectiveness of the methods.

Modified ant colony optimization algorithms used to balance the computing resources of unmanned aerial vehicles and the optimal length of their routes are already used for both individual [70] UAVs and for a group [71].

## 4 Experience of Ukraine

The creation of unmanned aircraft systems is one of the priorities of world civil aviation. Ukraine develops and manufactures helicopter-type UAVs, in particular « RS-1» (Ukrspetssistem LLC); «Khimera», «Commander» (OKB « Matrix of Technologies»). However, attempts to use them for mine detection continue at the research level [72].

«Ukrainian Multi-Motor Technologies » LLC (UMT) and researchers from the Binghamton University Geophysical Laboratory announced the integration of the Cicada UAV with the Geometrics MFAM atomic magnetometer system. The capabilities of high-resolution magnetic searches allow the use of Cicada-M in humanitarian demining operations, as an element of a broad technical survey that meets the provisions of international mine action standards (IMAS). Experiments on the identification of unexploded remnants of 122-mm artillery systems conducted at the Chernihiv airfield convincingly demonstrated that the implementation of such a geophysical system significantly reduces labour and time costs associated with technical assessment of polluted areas in the post-conflict region [73]. It took about 11 h to shoot and survey the area measuring 600 × 600 m. In the future, after synchronizing the frequency of the magnetometer with the GPS device, the duration of work is expected to be reduced to 3.5 h.

**Fig. 5:**
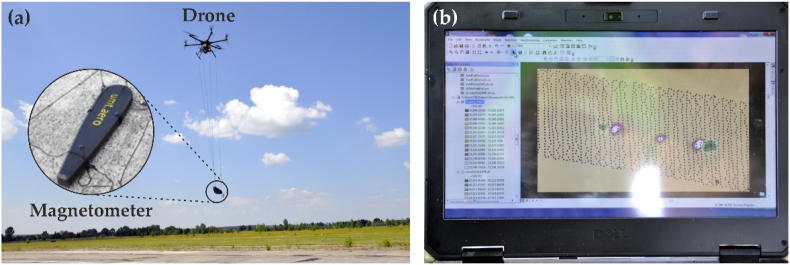
An unmanned aerial vehicle (UAV) with a magnetometer in the process of surveying the territory (a) and visualization and processing of spatial search data (b).

In January 2020, the State Research Institute for Testing and Certification of Weapons and Military Equipment announced the development of special magnetometer sensors for UAVs to detect unexploded ammunition by one of the domestic enterprises together with a foreign company [74]. The UAV equipped with such sensors operates at a height of 5–10 m and, in addition to locating explosive objects, identifies their contours (Fig. 5). The tests confirmed the unerring identification of mines of calibre 82 and 120 mm with centimetre accuracy. For such equipment, the period of the day does not matter, and the received information can be transmitted in real time, so the map of explosive objects is compiled quite quickly.

The dominant soils in Donbas seem to be generally suitable for shallow GPR [75]. The increased electrical conductivity due to the clay content may be the advantage of GPR rather than a limitation for shallow plastic (low conductivity) shells.

**Fig. 6:**
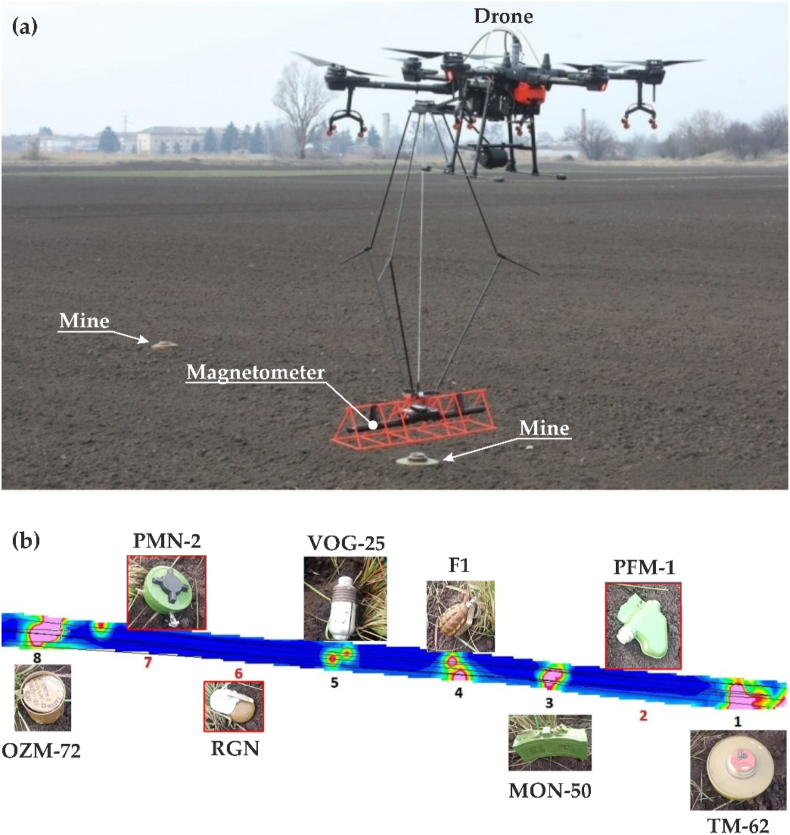
Mine detection using an unmanned aerial vehicles (UAV) equipped with a magnetometer during field tests (a) and the resulting map of the minefield with identified explosive objects and mines nearby (b) (according to Ref. [76]).

The Polish charitable foundation « Postup » created by natives of Ukraine, signed a memorandum of cooperation with the State Emergency Service and offers Ukrainian sappers the use of its own development – a magnetometric system combined with a UAV (Fig. 6). During the flight, the magnetometer sensor at a height of 30 cm above the surface continuously records the parameters of the Earth's magnetic field in a strip 2 m wide with accurate geopositioning. After landing, the information is processed in special software tools and forms a map of the minefield. The stated effectiveness of the system for an area of 240 m^2^ with ten mines, with the involvement of three sappers and two magnetometer operators will be 112 min. It was established that such a system cannot detect some types of mines with a small amount of metal parts (for instance, Russian PFM-1 and PFM-2), but it detects anti-tank mines at a depth of 50–60 cm (for instance, ТМ-62) [76].

**Fig. 7:**
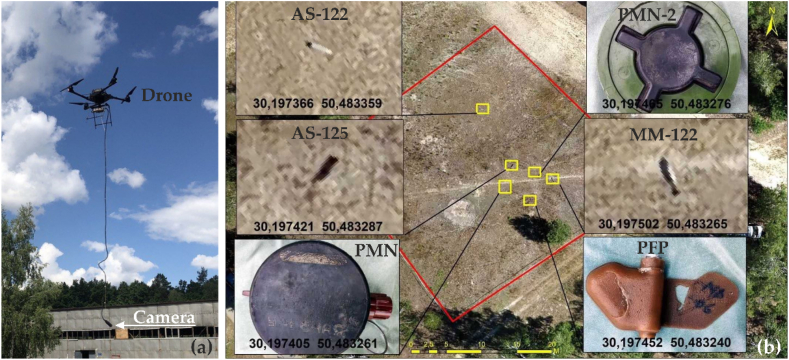
Detection of mines using an unmanned aerial vehicles (UAV) equipped with a magnetometer in the process of surveying the territory (a) and the results of identifying the location of mines and explosive objects (b) on the surveyed territory (according to Ref. [77]).

The Canadian company DraganFly and the Ukrainian Yellow-Blue Foundation presented their own program for demining territories using UAVs equipped with remote sensors that respond to the components of explosive objects at a depth of 3 m [77]. The sensors collect spatial information not only about the 3-D model of the terrain, the presence of metal objects, but also about their location at different values of the depth sections. Plastic objects are identified by the camera. The data collection process consists of several stages and processes divided between Ukraine and Canada. After flying over the area, the artificial intelligence software adapts the collected data to search for and detect different mines based on shape, size, material, type of explosive, etc. The data for comparison was developed by sappers in different countries who have experience with Russian and Soviet mines. As a result, 3-D maps of anomalies that may carry a mine threat are formed. The accuracy of detecting mines and explosive objects is close to 100%. An area of 1 ha takes 1 h, and the results are shown in Fig. 7.

Since 2020, the Scientific Centre for Aerospace Research of the Earth of the Institute of Geological Sciences of the National Academy of Sciences of Ukraine, as part of the project of the scientific and technical program “Research and development on the problems of increasing defence capability and security”, is developing a new technology for remote mine detection based on the automated analysis of multispectral survey materials from a helicopter-type UAV (quadracopter, multicopter). Classification of mines was developed with the use of special equipment for aerial photography: RGB-camera, IR camera, multispectral camera [78].

In June 2021, comprehensive field research on real objects began. The methodology of these tests, approved by the command of the Support Forces of the Armed Forces of Ukraine, provided for the preparatory stage, the conduct of pilot studies on the test areas of the test site together with the specialists of the Training Centre of Engineering Troops and the Troops of Radiation, Chemical, Biological protection, processing of the data obtained and evaluating the effectiveness of the proposed development.

Using the ASD FieldSpec 3FR spectrometer, precise optical reflection spectra of typical samples of metal and plastic mines (of various types and levels of camouflage) and spectral characteristics of the main classes of natural objects and surface textures of the area (green and dry grass, snow cover, water surface, etc.) were obtained. The research results were supplemented with thermal imager data using different levels of illumination. Photomosaics of multispectral and thermal (IR) images are classified separately and independently of each other. Multispectral data were processed by the method of binary logistic regression, and thermal data by a specialized algorithm. The pixel-by-pixel classification of digital mosaics by both methods was combined using a basic approach and a likelihood function with a threshold value that determined the presence/absence of a dangerous object was compiled.

The results of searching for real objects with different types of shooting were:

• multispectral – 92% (false alarm probability – 37%);
• infrared – 91% (false alarm probability – 45%);
• combined – 91,8% (false alarm probability – 41,6%).

The probability of correctly detecting mines is quite high and meets the global requirements to similar military systems, but for the purposes of humanitarian demining, the efficiency of the technology should be higher, which requires additional hardware improvement.

At present, the shortcomings identified as a result of tests are being eliminated, the result of which should be the production of a prototype on-board complex for remote sensing based on a UAV. In particular, it is proposed to supplement the on-board complex with a small-sized georadar, in addition to the multispectral and infrared cameras.

A separate direction is the development of semi-automated sapper robots whose activity is based on remotely active methods of geospatial data collection [16]. As part of NATO's Science for Peace and Security (SPS) program, in 2018, Ukrainian specialists, together with colleagues from Italy and the United States, created a U-GoFirst land-based robot sapper to search for roadside explosives, which uses pulse radar and 3-D surface scanning to detect landmines in real time [79].

In 2020, on the basis of the Igor Sikorsky Kyiv Polytechnic Institute, research work (0119U100703) was completed on the creation of a remote-controlled platform of a mobile demining robot with high cross-country ability and maneuverability [80]. On the basis of comprehensive research, for the first time, a combination of a ground remotely controlled chassis with an unmanned aerial system was proposed for the created airborne robotic complex, which can be used in various fields. In terms of cross-country ability and other parameters, the developed chassis corresponds to the best foreign designs and experimental samples («The New Chaos » USA in tracked and wheeled versions), and surpasses them in functionality and load capacity.

In August 2022, the express delivery leader Nova Poshta, in cooperation with the State Emergency Service of Ukraine, announced the development of sapper robots for demining water bodies and land, not only detecting mines, but also clearing them. The State Emergency Service determined the basic requirements for these robots, and Nova Poshta took responsibility for the technical direction, as well as for finding and attracting partners [81].

## 5 Discussion

Advanced classification and image recognition using machine learning/artificial intelligence has been used to identify mine probable areas. To improve the accuracy and reliability of AI detection of explosive objects in the visible range, it is first and foremost necessary to increase the library of source images. AI tools are able to generate additional images based on changes in angles, illumination, vegetation, and other specified characteristics.

One of the mine action groups regularly publishes open-source datasets and research, encouraging collaboration to improve existing mine action methods [82].

Ukraine intends to use all the technological tools at its disposal - from sophisticated artificial intelligence-based impact assessments to home-made unmanned aerial vehicles for mine detection - to speed up the traditionally slow and labour-intensive clearance process. The government is working with US data analytics giant Palantir to combine dozens of previously closed data streams and develop models that will determine which demining will have the greatest impact [83].

Related research, in particular [84], provides evidence of the state of the art - the availability of the required number of images, including those with rare foreign object anomalies, can be abnormal. Unique approaches using large-scale modeling approaches, such as ChatGPT (Generative Pre-trained Transformer) and text-to-picture models, allow increasing the number of foreign object images.

The absence and limited empirical data on the effectiveness of different basic models in detecting foreign objects form a platform for a relevant discussion. After all, each additional % of confidence can be expressed primarily in terms of the number of lives saved.

## 6 Conclusions

The use of UAVs in demining processes can be considered advanced and such that opens up the possibilities of existing and promising methods and technologies in a new way. A diverse range of models and structural features of modern UAVs allow them to be adapted to perform any tasks. The increased interest in UAVs creates trends for continuous improvement: the time spent in the air and the load capacity increase, navigation processes are optimized and accidents are minimized.

An insidious complication is experienced by new mines, the bodies of which are made of unusual materials (wood, plastic, etc.), they form a different composition of explosive substances, which changes the studied physical properties to the maximum and complicates the possibility of their further identification. Under such conditions, the use of only one known method for detecting mines and explosive substances becomes insufficient, and dramatically increases the likely risks and possible consequences. Therefore, depending on the conditions of the area and the means of damage used, a combination of at least several methods is justified.

It is obvious that the amount of information received from a UAV with each additional element of the payload will grow immeasurably, and therefore it must be properly structured for further analysis. A well-recommended solution under such conditions is the use of spatial databases and GIS-tools for their maintenance. The influence of many criteria on the search for a solution, often not at all obvious, requires a combination of the use of multi-agent systems and neural networks.

At the same time, the use of each of the technologies has its own peculiarities which should be considered during the preliminary collection of information. Thus, IR sensors are more suitable for locating a minefield (estimating the area of work), than for determining the position of individual mines from the air. Visual and hyperspectral cameras will always be lighter than GPR and metal detectors. Imaging systems in these ranges are often inexpensive, compact, have high spatial resolution, and can be used for real-time detection. The spectral response of landmines with plastic bodies has a different shape than the reflectivity of the background material. This gives hyperspectral imaging technology an advantage over well-known metal detectors in detecting plastic mines, which are the vast majority today. The utility of using radar will depend primarily on the resolution of the measurements. Using only one wavelength range is dangerous, as there is a possibility that meteorological conditions will have a strong influence on the accuracy of the measurements.

The experience of Ukraine in humanitarian demining using innovative methods can be considered initial, but one that is actively developing at the stage of testing experimental prototypes. Its tendencies suggest a remote minimal participation of a human operator, who semi-automatically uses robotic systems in the form of land-based drones or helicopter-type UAVs. Considerable emphasis is placed on electromagnetic and remote sensing methods. In total, their established performance already exceeds 90%, but it is far from the necessary one specified in the standards for humanitarian demining. Thus, at the same time, a search is underway for ways to combine different types of methods with the involvement of advanced intelligent methods for sensor data processing and spatial information analysis.

## Data availability statement

Data included in article/supp. material/referenced in article.

## Ethics statement

Review and/or approval by an ethics committee was not needed for this study because this work is a review of the literature and does not address the ethical considerations of animal, cell, and human experimentation.

## CRediT authorship contribution statement

**T. Hutsul:** Writing – review & editing, Writing – original draft, Visualization, Validation, Methodology, Investigation, Data curation, Conceptualization. **M. Khobzei:** Writing – original draft, Investigation. **V. Tkach:** Writing – original draft, Visualization. **O. Krulikovskyi:** Writing – original draft, Investigation. **O. Moisiuk:** Writing – original draft. **V. Ivashko:** Writing – original draft, Investigation, Data curation. **A. Samila:** Writing – review & editing, Writing – original draft, Visualization, Validation, Supervision, Methodology, Investigation, Conceptualization.

## Declaration of competing interest

The authors declare that they have no known competing financial interests or personal relationships that could have appeared to influence the work reported in this manuscript.

## Abbreviations

3-D: 3-dimensional
5G: fifth-generation technology standard for cellular networks
AI: artificial intelligence
CNN: сonvolutional neural network
DDRQN: double deep recurrent Q-network
DSS: a decision support system
GIS: geographic information systems
GNSS: Global Navigation Satellite System
GPR: ground penetrating radar, geo-penetrating radar systems
GPS: Global Positioning System
EM: Electromagnetic
IMAS: International Mine Action Standards
IoT: Internet of things
IMU: an inertial measurement unit
IR: infrared
LiDAR: light detection and ranging
MOMS: multi-optical mine detection system
NQR: nuclear quadruple resonance
RGB: red, green, blue colour model
R–CNN: Region-based Convolutional Neural Network
SAR: synthesized array radar
SDR: a software-defined radio
UN: United Nations
UAV: unmanned aerial vehicles
